# The 6th R of Radiobiology: Reactivation of Anti-Tumor Immune Response

**DOI:** 10.3390/cancers11060860

**Published:** 2019-06-20

**Authors:** Jihane Boustani, Mathieu Grapin, Pierre-Antoine Laurent, Lionel Apetoh, Céline Mirjolet

**Affiliations:** 1 Department of Radiation Oncology, Unicancer-Georges-Francois Leclerc Cancer Center, 21079 Dijon, France; jboustani@cgfl.fr (J.B.); mgrapin@cgfl.fr (M.G.); palaurent@cgfl.fr (P.-A.L.); 2 INSERM, U1231 Dijon, France; lionel.apetoh@inserm.fr

**Keywords:** radiotherapy fractionation, immune response, radiotherapy-immunotherapy association

## Abstract

Historically, the 4Rs and then the 5Rs of radiobiology explained the effect of radiation therapy (RT) fractionation on the treatment efficacy. These 5Rs are: Repair, Redistribution, Reoxygenation, Repopulation and, more recently, intrinsic Radiosensitivity. Advances in radiobiology have demonstrated that RT is able to modify the tumor micro environment (TME) and to induce a local and systemic (abscopal effect) immune response. Conversely, RT is able to increase some immunosuppressive barriers, which can lead to tumor radioresistance. Fractionation and dose can affect the immunomodulatory properties of RT. Here, we review how fractionation, dose and timing shape the RT-induced anti-tumor immune response and the therapeutic effect of RT. We discuss how immunomodulators targeting immune checkpoint inhibitors and the cGAS/STING (cyclic GMP-AMP Synthase/Stimulator of Interferon Genes) pathway can be successfully combined with RT. We then review current trials evaluating the RT/Immunotherapy combination efficacy and suggest new innovative associations of RT with immunotherapies currently used in clinic or in development with strategic schedule administration (fractionation, dose, and timing) to reverse immune-related radioresistance. Overall, our work will present the existing evidence supporting the claim that the reactivation of the anti-tumor immune response can be regarded as the 6th R of Radiobiology.

## 1. Introduction

The generation of an immune response to eliminate cancer cells requires several steps, the first one being the capture and processing of tumor associated antigens (TAA) by dendritic cells (DCs) for processing [1,2]. DCs present TAA on MHC molecules to T cells, resulting in the priming and activation of tumor-specific naive T cells that become effector T cells. The activated effector T cells traffic to tumor tissues where they infiltrate the tumor bed and kill target tumor cells. Tumor cell lysis leads to TAA release, leading to subsequent striking changes in the immune response.

Historically, radiotherapy (RT) was considered as an immunosuppressive treatment due to its role in the preparation for the allogeneic transplant through total body irradiation. Current evidence has demonstrated that besides its direct action on tumor cell DNA, RT can induce systemic and immune-mediated anti-tumor responses.

### Radiation Therapy Effects: From DNA Breaks to Immunomodulation

Conventional anti-cancer treatments such as RT or chemotherapy exert a direct anti-tumor effect through DNA damage. The direct cytotoxic effect of ionizing radiation is the result of three successive phases (physical, chemical, and cellular). Radiation creates highly reactive radicals from water molecules responsible for DNA lesions. This results in cellular lesions, whose occurrence depends on the DNA repair system. A significant amount of damage within malignant cells ultimately leads to the loss of clonogenicity, induction of cell death and finally reduction of tumor size. The most important biological factors influencing tumor and normal tissue responses to fractionated treatment are referred to as the “five Rs” [3,4]:

- Repair: the capacity to repair radiation-induced DNA damage.
- Re-assortment: cells in mitosis are mostly sensitive to radiation, whereas cells in S-phase are mostly resistant. With multiple doses, cells progress through to a sensitive phase of the cell cycle, which leads to the therapeutic effect.
- Repopulation: cell proliferation between fractions.
- Reoxygenation: the sensitivity to radiation increases in well oxygenated tissues.
- Radiosensitivity: the response to radiation varies by tumor intrinsic and individual radiosensitivity.

**Figure 1:**
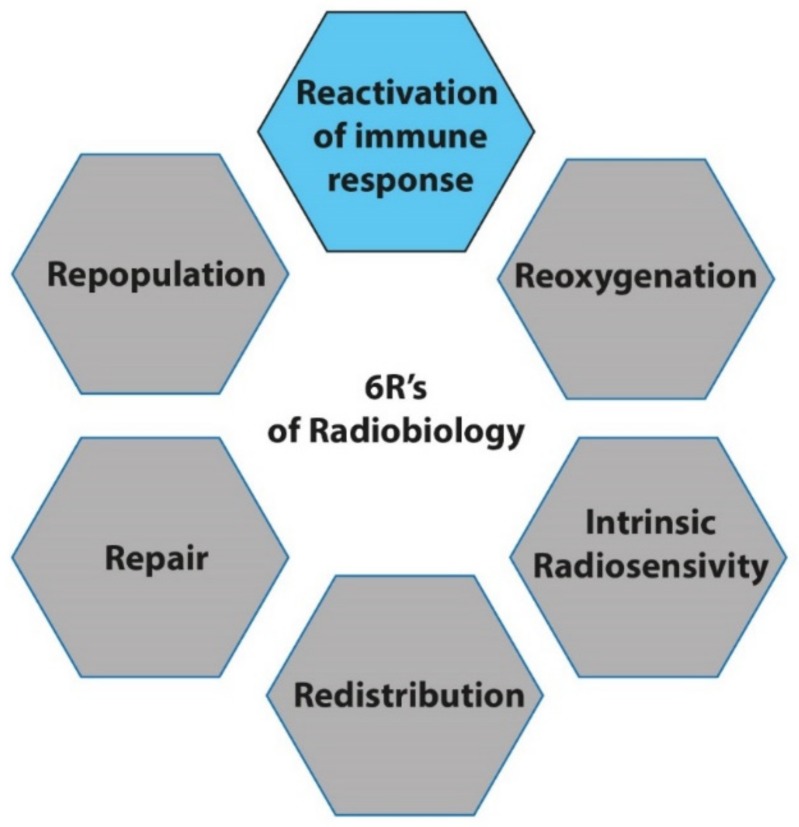
The reactivation of the immune response: the new R of radiobiology.

With the advent of immunotherapy in cancer treatment, we propose a 6th R that stands for the Reactivation of the immune system by RT (Figure 1). This topic will be further discussed below.

The exposure to RT induces the activation of a multicomponent signal transduction network, known as DNA damage repair (DDR) [5]. These DNA alterations facilitate oncogenesis but also alter the tumor microenvironment (TME) and the inflammatory cascade [6]. DNA damage repair pathways are not limited to post-RT, but are also part of some hereditary syndromes and oncogenesis [7]. RT and chemotherapy exploit DDR to target cancer cells [8]. The radioresistance of a cell line (such as PC-3, patient prostate cancer cells) can be explained by the up-regulation of DDR genes (BRCA1, FANCG, RAD51) to promote cell survival following irradiation [9].

The innate immune system can recognize molecular patterns associated to both extracellular and intracellular pathogens using pattern recognition receptors (PRR) such as Toll-like receptors, NOD-like receptors, and C-type lectin receptors. It is now clear that PRRs can be directly regulated by the DNA damage response. ATM, a kinase activated in response to DNA double-strand breaks, can regulate PRR activation and interferon signaling [10]. 

A role for T cells in the tumor response to radiation was first suggested by Stone et al. in experiments demonstrating a reduced therapeutic efficacy in irradiated mice that lacked a normal T-cell repertoire [11]. Since then, many studies and reviews have led to a better understanding of the relationship between anti-tumor immunity and RT [12,13]. It is now well known that RT can result in immunogenic cell death (ICD) by inducing the cell surface translocation of calreticulin, the extracellular release of the high-mobility group protein B1 (HMGB1) and the release of adenosine triphosphate (ATP) [14]. These events are critical for DC activation and effector T cell priming. Additionally, RT promotes the cytotoxic CD8 and Th1 cells recruitment to TME by the induction of chemokines such as CXCL9, CXCL10 and CXCL16 [15], as well as cell adhesion molecules such as the intercellular adhesion molecule (ICAM)-1 and vascular cell adhesion molecule (VCAM)-1. These molecules mediate the lymphocyte adhesion to the vascular endothelium [16]. Proinflammatory cytokines such as interleukin 1*β* [17], tumor necrosis factor α [18] and type 1 and 2 interferons [19] can also be induced by radiation. Moreover, RT can counteract tumor immune evasion by upregulating MHC class 1 molecules on the surface of tumor cells and by modulating their peptide repertoire, allowing tumor cell recognition by cytotoxic CD8 T cells [20,21]. In addition, the expression of T cell co-stimulatory molecules CD80/CD86 can be induced on the surface of tumor cells after irradiation [22]. RT also increases the expression of NKG2D receptor stress ligands, activating tumor cell clearance by Natural Killer (NK) cells [23].

Overall, RT induces modifications that contribute to an increase in the ability of tumor cells to be recognized by the immune system and to the activation of both innate and adaptive immunity effectors that contribute to a specific anti-tumor immune response. The irradiated tumor becomes a real in situ vaccine. In addition, a systemic anti-tumor effect of local RT has been described. The regression of a metastasis outside the irradiated field, known as the abscopal effect, best demonstrates the existence of such a systemic response [24]. This phenomenon was described in animal models and in some patients [25]. On the other hand, RT was shown to promote immunosuppressive mechanisms, such as increasing regulatory T cells (Tregs), TME infiltration by myeloid-derived suppressor cells (MDSCs) [26] and the development of pro-tumor tolerant type 2 macrophages [27,28], hence limiting the effectiveness of anti-tumor immune responses [29]. Finally, RT can increase the expression of Programmed Death Ligand 1 (PD-L1) on the surface of tumor and immunosuppressive myeloid cells [30] as well as the expression of the T cell immunoreceptor with Ig and ITIM domains (TIGIT), a co-inhibitory receptor expressed on CD8+ T cells, natural killer cells, Tregs and T follicular helper cell [31,32,33]. TIGIT is a transmembrane glycoprotein receptor with an Ig-like V-type domain and an ITIM in its cytoplasmic domain. The TIGIT ligands, CD155 and CD112 can be expressed by different cell types, including antigen-presenting cells and tumor cells [34,35]. TIGIT is associated with CD8+ T cell dysfunction [36]. The effectiveness of the radiation-induced anti-tumor immune response depends on the balance between immunostimulatory and immunosuppressive effects, which may be dependent on the RT fractionation schedule. However, RT alone is not sufficient to induce a strong long-lasting systemic immune response. These data support a treatment combination to overcome immunosuppressive mechanisms. Preclinical and clinical studies have demonstrated improved outcomes when combining RT with various types of immunotherapy, in particular with immune checkpoint inhibitors (ICI).

## 2. Overcoming Radiation-Induced Immune Resistance

### 2.1. Modulation of Radiation Therapy

#### 2.1.1. Finding the Optimal RT Dose and Fractionation to Induce Anti-Tumor Immune Response

Advances in RT (image-guided RT, intensity-modulated RT and stereotactic body RT (SBRT)) allow the delivery of moderate to high doses per fraction without increasing side effects. In clinical studies, a higher biological effective dose (BED) was associated with an improved local control in different tumor types. A meta-analysis of pre-clinical studies showed that the frequency of the abscopal effect increased with the BED, with a probability of revealing an abscopal effect of 50% when a BED of 60 Gy was achieved [37]. However, BED is an in vitro concept that does not take into account the impact of the TME, a crucial factor in the anti-tumor immune response. Thus, a practical model for immuno-RT was proposed, taking into account the immunologically effective dose [38]. Nevertheless, to methodically compare the immune effect of different RT doses per fraction schemes, we would need to use RT schemes with the same BED, which is not always done [19]. Recently, a threshold dose leading to effective anti-tumor immunity (≤ 8–10 Gy) was suggested in a preclinical setting [39]. Achieving the best anti-tumor response in terms of strength and duration requires the set-up of the ideal RT regimen (dose, fractionation and timing), which should be associated to the ideal immunotherapy.

**Figure 2:**
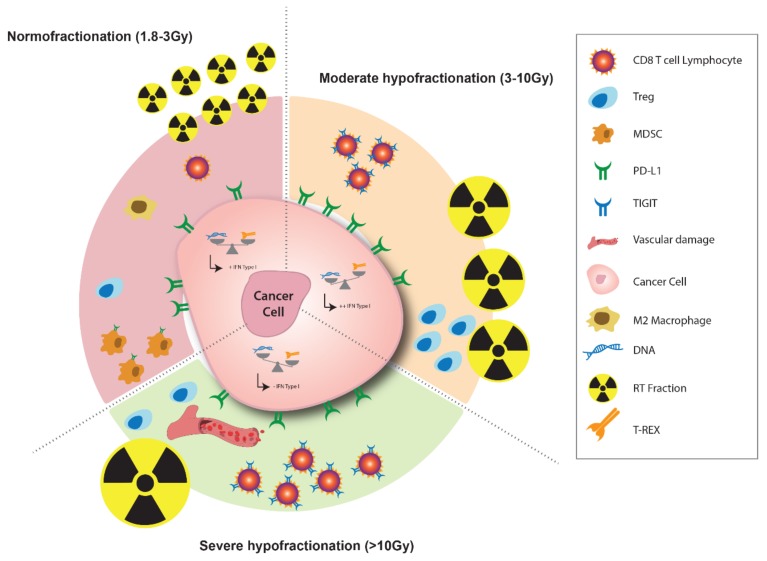
Radiotherapy in dependence of the fractionation schedule recruits different type of immune cells and can modulate the immunotherapy target expression. (Treg: regulatory T Cell; MDSC: Myeloid-derived suppressor cells; PD-L1: Programmed-Death Ligand 1; TIGIT: T-Cell immunoreceptor with Ig and ITIM domain; DNA: Desoxyribonucleic Acid; RT: Radiotherapy; T-REX: Three-Prime Repair Exonuclease). These results spring from Vanpouille-Box et al., Nature Com, 2017 and Grapin et al., JITC, in press.

Vanpouille-Box et al. have shown in a pre-clinical model that doses per fraction greater than 12 Gy reduced the immunogenicity of different cancer cells and the abscopal effect. Further investigations revealed that these observations were related to the cytoplasmic accumulation of an exonuclease called Three-Prime Repair exonuclease 1 (TREX1), which degrades accumulating cytosolic DNA following irradiation [39]. Why does this prevent the elicitation of anticancer immunity? This is because cytosolic DNA was recently coined as a danger signal due to its ability to bind to cyclic GMP-AMP synthase (cGAS) and activate the adaptor protein Stimulator of Interferon Genes (STING) [40,41,42]. Upon binding to cGAS, double-stranded DNA induces the formation of a cyclic GMP–AMP (cGAMP) that activates STING, leading to the engagement of intracellular signaling pathways, culminating in the induction of type I interferons and proinflammatory cytokines [43,44]. Thus, when cytosolic DNA is present, it stimulates the interferon β secretion through STING, and drives the DC recruitment and subsequent activation of anti-tumor immune responses. Therefore, observations that upon RT treatment the cytosolic DNA concentration gradually increases to a dose of 12 Gy per fraction and then collapses for higher doses suggest a form of radioresistance. The latter correlates with an increase in immunosuppression and the progressive loss of the abscopal effect. In many tumor models, including TSA and MCA38, the use of a single large radiation dose of 20 to 30 Gy reduced the tumor immunogenicity and loss of the abscopal effect compared to a moderate and repeated fractionated regimen such a 3 × 8 Gy [39]. High doses per fraction have been found to increase tumor infiltration by pro-tumorigenic macrophages [27]. Moreover, doses per fraction > 10 Gy induce significant vascular damage and reduce the vascular flow. Indeed, endothelial cell death leads to a reduced tumor effector T-cell recruitment [45]. The preclinical data suggest that the anti-tumor immune response is more important with hypofractionated regimens, with a dose threshold beyond which the immune and vascular factors leading to radioresistance are increased. A mathematical model suggested that the optimal radiation doses per fraction maximizing anti-tumor immunity are between 10 and 13 Gy [46]. However, the relation between the dose and immunity is complex and needs to take into account the total dose and the overall treatment time [37]. A recent pre-clinical study compared three fractionated protocols with the same BED: 1 × 16.4 Gy, 3 × 8 Gy and 18 × 2 Gy. In the absence of an immune checkpoint inhibitor, 3 × 8 Gy and 1 × 16.4 Gy induced a strong lymphoid response (regulatory T cells and CD8+ T cells), 1 week after RT initiation. An 18 × 2 Gy schedule induced a persistent myeloid cell increase (MDSC, TAM 2), 2 weeks after RT initiation [33] (Figure 2). 

To date, no clinical studies have directly compared different dose-fractionation regimens regarding their abscopal effect or immunogenicity. An immunosuppressive effect of conventional RT, delivering 1.8–2 Gy per fraction over several weeks, was suggested using a mathematical model. These data are consistent with treatment-related lymphopenia commonly seen in irradiated patients [37]. A study comparing SBRT to conventional fractionation RT in pancreatic cancer, revealed that the rates of lymphopenia were 13.8% versus 71.7%, respectively [47]. Moreover, macrophages receiving low doses (0.1–0.5 Gy) exhibit an immunosuppressive status, namely showing reduced IL-1β and increased TGF-β [48]. In an in vitro model, normofractionated RT induced TGF-β and IFN-related genes, leading to an immunosuppressive TME [49]. On the other hand, normofractionated RT could have a positive effect on TME, such as normalizing the tumor vasculature and facilitating immune cell migration across the endothelium [50], or inducing the M1 macrophage phenotype [50,51]. In fact, several studies have demonstrated the M2 polarization after a treatment with single high-dose and hypofractionated radiation regimens [28,52]. Additionally, RT is known to induce Tregs, which are radioresistant [53] and shift the balance of T lymphocytes [54]. This effect seems to be reduced in the context of moderate hypofractionation [55,56]. Finally, the effect of dose fractionation on MDSC is still unknown. The infiltration of MDSC into TME has been reported after both single high-dose and conventional fractionation regimens, often associated with a TAM infiltration [26,57]. Hypofractionated RT could decrease the number of MDSC in peripheral blood [58,59]. Understanding the different effects of conventional fractionation versus hypo-fractionation on the immune system may allow the improvement of combination therapies in a personalized medicine setting [60].

In a different context, low-dose X-irradiation therapy has been described as exerting an anti-inflammatory effect on benign diseases and chronic degenerative disorders with a maximum effect in the range of 0.3 to 0.7 Gy [61].

#### 2.1.2. The Immunoregulatory Potential of Particle Radiation: Hadrontherapy versus Proton Therapy

The effect of heavy ion RT on cell death signaling may elicit a more generalized immune response, compared with the one observed with photon-based therapy [62,63]. Lupu-plesu et al. showed, in head and neck squamous cell carcinoma, that (lymph)angiogenesis and inflammation genes were down-regulated (except for *vegf-c*) after proton irradiation and up-regulated after photon irradiation in surviving cells, demonstrating a more favourable profile with proton irradiation [64]. A retrospective comparison of patients with head and neck tumors treated with protons or carbon ions revealed a similar local control but a higher overall survival in patients treated with carbon ions compared to protons, which could be explained by an increased immune response [65]. The connection between heavy particles and immunity is still unclear, and preclinical studies are ongoing.

### 2.2. Combination of RT and Immune Checkpoint Inhibitors

#### 2.2.1. Anti-CTLA-4, PD-1 and PD-L1

Immune checkpoints are promising targets for enhancing a radiation-induced immune response. Cytotoxic T Lymphocyte Antigen-4 (CTLA-4) and Programmed cell Death-1 (PD-1) are expressed on T lymphocytes as co-inhibitory molecules that regulate T cell activation, leading to dysfunction [66,67]. In immune cells associated with the tumor and the TME, CTLA-4 and PD-1 are up-regulated, leading to an immune tolerance toward cancer. Moreover, as previously mentioned, RT can upregulate PD-L1 on tumor and immune cells in the TME. Antibody antagonists targeting CTLA-4 and PD-1/PD-L1 attenuate the tumor-induced inhibitory signaling, thereby shifting toward T-cell stimulation [66].

Demaria et al. first reported a synergy between the RT and CTLA-4 blockade in a preclinical setting [68], showing that the combination of radiation with an anti-CTLA-4 antibody, but not either treatment alone, improved the local control of the irradiated tumor and inhibited irradiated lung metastases growth. Furthermore, a fractionated regimen of 3 × 8 Gy, given on consecutive days, was the most effective, while a single large dose of 20 Gy was unable to induce an abscopal effect in combination with anti-CTLA-4. In the clinical setting, the CTLA-4 blockade was the first to show an overall survival benefit in patients with metastatic or unresectable melanoma [69]. In the phase 3 CA184-043 trial, patients with metastatic castration-resistant prostate cancer received bone-directed RT (1 × 8 Gy, up to 5 lesions) and were then randomly assigned 1:1 to either an anti-CTLA-4 (ipilimumab) or placebo. An RT combination with ipilimumab significantly prolonged a progression-free survival (HR = 0.70; *p* < 0.0001) [70].

Several preclinical models have demonstrated an increased effect when an RT and PD-1/PD-L1 blockade were combined [71,72]. Moreover, clinical studies demonstrated that the combination of local irradiation with the anti-PD-1/PD-L1 checkpoint blockade was a synergistic anti-cancer treatment. The PACIFIC trial found significantly longer PFS and OS in patients with stage III non-small cell lung cancer (NSCLC) treated with durvalumab, an anti-PD-L1 antibody, compared to a placebo after a concurrent chemoradiotherapy [73]. The 24-month OS rate was 66.3% in the durvalumab group, as compared with 55.6% in the placebo group (*p* = 0.005). In another clinical trial, 25% of patients with melanoma showed a regression of non-irradiated lesions when anti-PD-1 was continued after RT on a tumor site that had progressed upon anti-PD-1 monotherapy [74].

#### 2.2.2. Other Immunotherapies

Anti-TGFβ: RT triggers the activation of TGFβ, an immunosuppressive cytokine, which in turn promotes DNA damage repair and impairs the antigen-presenting function of DCs, and the functional differentiation of T cells into effectors [75,76]. Vanpouille-Box et al. have shown that anti-TGFβ antibodies administered during RT uncover the ability of RT to induce T cell responses to endogenous tumor antigens in a pre-clinical model of metastatic breast cancer [77]. Importantly, only the combination of RT with anti-TGFβ, but not each treatment alone, induced the T cell-mediated rejection of the irradiated tumor and non-irradiated metastases in mice, indicating that blocking TGFβ unleashes the potential of RT to generate an in situ tumor vaccine [77]. A prospective randomized trial was subsequently conducted in metastatic breast cancer patients with at least three distinct metastatic sites [78]. One metastatic site received a conformal RT of 3 fractions of 7.5 Gy in combination with a TGFβ blocking antibody, fresolimumab. Two doses of fresolisumab were compared. This study showed that a TGFβ blockade during RT was feasible and well tolerated. Patients receiving the higher fresolimumab dose had a favorable systemic immune response and experienced longer median OS than the lower dose group [78].

Anti-TIM-3: T cell immunoglobulin mucin–3 (TIM-3), a negative immune regulator that is well known as a dysfunction marker of CD4 and CD8 positive lymphocytes, could also be targeted. Indeed, the anti-tumor efficacy of anti-TIM-3 has been demonstrated in a pre-clinical model of murine glioblastoma using the combination of anti-PD-1 blockade and focal RT [79], as well as in other models in combination with different immune-checkpoint inhibitors or alone [80]. The blockade of TIM-3, which enhances T-cell cytotoxicity and decreases Tregs, showed a synergistic effect in association with RT [81].

Anti-TAM2: In a mouse model, some authors described an anti-tumor effect when combining antibodies against M2 macrophages (anti-M-CSF and anti-F4/80) with RT [52].

Anti-TIGIT: We have recently investigated, in the context of RT, the relevance of modulating the TIGIT signaling. For this, two fractionation protocols with the same BED (18 × 2 Gy and 3 × 8 Gy) were compared, and we found that 3 × 8 Gy increased the expression of TIGIT on CD8+ T cells (Figure 2). Driven by these observations, we have tested the therapeutic efficacy of a treatment combining anti-TIGIT, anti-PD-L1, and fractionated RT (3 × 8 Gy). We found that this combination led to an impressive anti-tumor response, with 9/10 mice achieving a complete response. This anti-tumor effect was not found with the other RT treatment regimen (18 × 2 Gy), consistent with the reduced TIGIT expression with a normofractionated schedule [33].

### 2.3. Optimization of RT-Immunotherapy Combination

A major challenge in combining RT with immunotherapy is to identify not only the optimal molecule and the best radiation regimen, but also to determine the best chronological sequence for their application. Currently, there is no established consensus on the optimal timing in the clinical setting. The available data tend towards a concurrent combination. Dovedi et al. have shown in mice bearing CT26 colorectal cancer that a PD-L1 blockade was only effective when given concurrently (day 1 or day 5) with fractionated RT (5 × 2 Gy), but not sequentially one week after [82]. Similarly, in a murine breast cancer model, an anti-CTLA-4 antibody given 2 days before or on the day of irradiation, achieved better results than a delayed administration 2 days after irradiation [83]. In the phase 1 KEYNOTE 001 trial, RT prior to a pembrolizumab treatment in patients with advanced NSCLC resulted in a longer PFS and OS, when compared to patients who did not receive prior RT, with an acceptable safety profile [84] (p001). A subgroup analysis of the PACIFIC trial showed that PFS was higher when patients received durvalumab within 14 days after chemoradiation, compared to those who received treatment > 14 days after (hazard ratio of 0.39 vs. 0.63) [73]. Furthermore, these data suggest a possible benefit when RT precedes the PD-1/PD-L1 blockade. Given that the PD-L1 expression on tumor and immune cells is upregulated after radiation and serves as a mechanism of resistance by promoting T-cell dysfunction, the inhibition of the PD-1/PD-L1 axis shortly after RT seems reasonable.

In addition, the ideal timing of administration depends on the mechanism of action of the immunotherapy. In Young et al., CT26 colorectal carcinoma bearing mice were treated with 20 Gy radiation combined with either anti-CTLA-4 antibody or OX40 agonist antibody [85]. Anti-CTLA-4 was most effective when given prior to RT, while the OX40 agonist antibody was optimal when delivered one day after [85]. These findings are consistent with known mechanisms of CTLA-4 and OX40 therapies; the CTLA-4 blockade can deplete intratumoral Tregs prior to RT to mitigate the immunosuppressive TME, while OX40 co-stimulatory molecules are upregulated only for a brief period after the antigen presentation induced by the radiation [86,87].

Based on the available data, an immunotherapy administration prior to or at the time of RT might be more effective to overcome immune resistance and reinvigorate the anti-tumor immune response. However, further studies are required to define the best timing, as well as the potential added toxicities.

The optimization of an RT-Immunotherapy combination should also include RT optimal dose and fractionation parameters. As previously described, the RT dose and fractionation scheme can influence its modulatory effect on the immune system. A high-dose RT appears to elicit a favourable tumor immunogenic modulation. A repeated exposure to RT may eliminate resident and infiltrating lymphocytes, negating any beneficial therapeutic effects. Hence, due to the shorter time of delivery of a high-dose regimen, it may avoid lymphocyte eradication. Interestingly, Serre et al. proposed an explanation for this differential effect based on the concept of the Immunologically Effective Dose (IED), which models the intrinsic immunogenicity of RT schedules [38]. Despite having a similar BED, the two schedules differed by their IED. Indeed, the IED efficacy of 3 × 8 Gy was more than twice the IED efficacy of the 5 × 6 Gy regimen.

Each fractionation protocol modulates the immune landscape differently and the immune checkpoint more precisely. Hence, each fractionation protocol would need a specific immunotherapy to reverse the immunosuppression. Normofractionated schedules would need an anti-PD-L1 consistent with the fact that repeated fractionations induce the expression of PD-L1 [82]. Based on our current findings, we speculate that moderate hypofractionated schedules might need an anti-TIGIT and an anti-PD-L1 [33]. 

## 3. Prospects About Associating Focal Irradiation with Biotherapies Targeting Recently Discovered Immune Pathways: Toward a Promising New Weapon in the Anti-Cancer Armamentarium?

As previously described, combinations between RT and immune-checkpoint blockades/agonists are currently under investigation, aspiring to become a standard of care in a foreseeable future. We believe that the optimization of the RT fractionation schedule for each type of immunotherapy and/or tumor localization is required to achieve strong anti-tumor responses. Moreover, novel immune checkpoint targets in pharmaceutical company pipelines are currently being evaluated alone or in association with chemotherapy. We are strongly interested in developing early-phase clinical research on the optimization of their potential association with RT. However, the following three prerequisites are required to consider this approach:

- Simultaneous research on the optimal fractionation and best RT modalities to efficiently promote the action of a concomitantly-administered drug targeting immune checkpoints. These conditions should differ depending on the drug that is used, and careful reevaluation should be done for each novel molecule.
- Integrating, in early-phase clinical trials investigating novel immune-checkpoint blockers under development in pharmaceutical pipelines, their association to RT, before their approval by medical drugs agencies such as the Food and Drug Administration (FDA) in the United States or the European Medicines Agency (EMA) in Europe. This should contribute to their optimal use once marketed.
- The development of clinical and biological tools that can be used routinely to help clinicians identify the patients most likely to benefit from a therapeutic scheme combining new ICI and RT and to help them decide on the most appropriate RT schemes to be used.

### 3.1. Association of Specific Chemotherapy with RT and Immunotherapy

**Figure 3:**
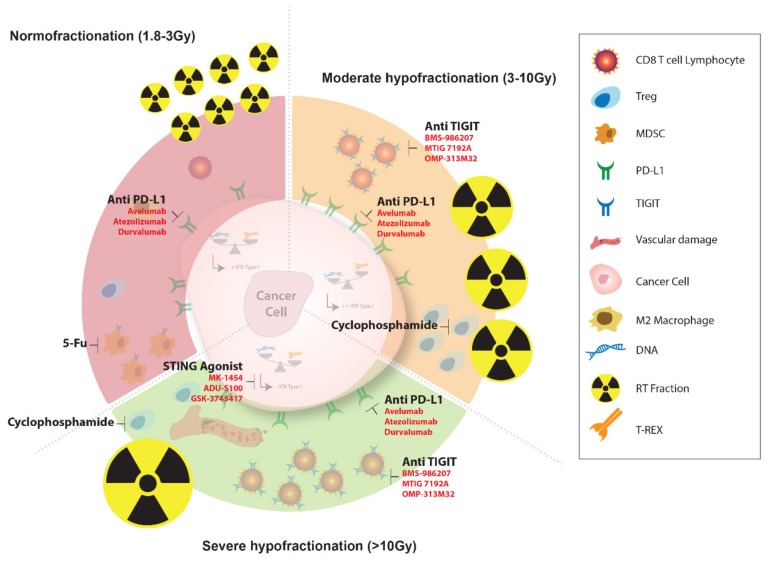
According to the immune response induced by three types of fractionation schedules, we suggest optimized radio-chemo-immunotherapy protocols.

As previously described, RT has negative implications with regards to immune system activation, some of which might be thwarted by adding concomitant and/or sequential chemotherapeutic agents. Considering the perspective of killing MDSC as a key-issue, an association between 5-FU-based chemotherapy, known for its ability to efficiently target MDSC [88], and RT delivered with a dose and a fractionation schedule that is more likely capable of inducing MDSC in TME, could be evaluated. Moreover, as described earlier, Tregs, which are significantly increased after RT when compared to other T-cell subpopulations, can be preferentially targeted by a wider use of chemotherapeutic drugs in addition to focal RT. For example, a significant part of the immune-potentiating effects of cyclophosphamide comprises Tregs selective depletion [89], although this drug is under-used in RT-associated regimens in current clinical practice (Figure 3). We suggest performing early-phase clinical trials evaluating RT with concurrent cyclophosphamide, for instance in association with SBRT in the management of oligometastatic breast cancer.

### 3.2. Association of Extreme Hypofractionated RT with STING Agonist

The STING pathway, which has already been described earlier in this article, appears to be a promising target. Deng et al. [44] showed that innate immune sensing following RT was predominantly mediated by a STING-dependent mechanism and that adding cGAMP, a STING agonist, significantly reduced the radioresistance and enhanced the anti-tumor immune responses. Pursuing the same objective, new STING agonists are currently being tested in a clinical phase I study, such as MK-1454, which is evaluated in combination with concomitant anti-PD-L1 antibody pembrolizumab [90]. However, none of these STING agonists have not yet been explored in association with RT. Moreover, TREX-1 appears to be an interesting target as well, since its activation in tumor cells was modified by a single-fraction delivered RT in a dose-dependent regimen [91] (Figure 3). Taking these data into consideration, an early-phase clinical trial testing a possible association between a hypofractionated RT in the treatment of liver metastases of a colorectal cancer and STING agonists seems challenging, as this association might counterbalance the deleterious activation of TREX-1 exonuclease.

### 3.3. TIM-3 and TIGIT: A Contemporary Lack of Data Leading Toward a Promising Future?

In two different models of murine lung adenocarcinoma, TIM-3 was upregulated in the context of resistance to anti-PD-1 therapy. A significant survival advantage was observed with the addition of a TIM-3 blocking antibody following a failure to PD-1 blockade [92]. Therefore, understanding how RT can upregulate the TIM-3 expression on TILs in different models according to several irradiation schedules represents a major challenge in preclinical investigations, which is currently far from being achieved. In the near future, studying the association of an anti-TIM-3 antibody and a metastasis-directed SBRT with an anti-PD-1 systemic treatment in patients with a metastatic NSCLC resistant to PD-1 blockade may be relevant.

Likewise, TIGIT, whose expression can be increased by focal RT in a syngenic model of murine colorectal cancer depending on the fractionation, particularly with a 3 × 8 Gy schedule [33], and which can already be targeted by optimized antibodies, seems to be a perfect pathway to modulate in combination with RT alone [93] or with other ICIs, such as those targeting TIM-3 [94] or PD-1 [95].

### 3.4. Combination Treatments Associating RT with ICI-Based Immunotherapy: What If Personalization Was the Keyword?

Finally, RT might play a major role in the huge challenge represented by the heterogeneity of therapeutic responses in patients treated with ICI-based regimens. However, this encouraging perspective is conditioned by the knowledge of the optimal irradiation scheme to propose to the right patient capable of benefiting from it, according to intrinsic characteristics of its tumor and its predictable immunogenicity.

This personalized approach to combination treatments including RT must be based on the use in current clinical practice of tools currently confined to preclinical research, capable of predicting the targets inducible by RT and potentially accessible to the addition of concomitant ICI-based immunotherapy, as well as which RT regimen seems the best suited to induce these targets.

With this in mind, Vanpouille-Box et al. [96] developed the concept of patient-derived xenografts (PDX), directly obtained from diagnostic biopsies, transplanted on immunocompromised mice and subsequently irradiated with different RT schedules to evaluate the intra-tumor immune response and the modulation of the ICI target expression. The analysis of the aforementioned pathways in the transplanted tissue should constitute a key pillar in treatment personalization.

## 4. Conclusions

RT considerably modifies the immune landscape by affecting immune activation as well as immunosuppressive pathways. The RT regimen, and more precisely the dose per fraction and consecutive fractions, have a real impact on this immune regulation. That is why the impact of RT fractionation on immune response activation can justify the addition of the 6th R of radiobiology, which represents the “Reactivation of anti-tumor immune response”.

In the era of immunotherapy, identifying how each RT protocol increases immunosuppressive barriers is necessary to design and implement synergistic drug therapies targeting multiple pathways. Here, we suggest that there is no unique immunogenic RT regimen, but rather an immunogenic effect resulting from the combination of a specific RT regimen and a specific immunotherapy.
